# On the role of exploitation and exploration strategies in the maintenance of cognitive biases: Beyond the pursuit of instrumental rewards

**DOI:** 10.3758/s13421-023-01393-8

**Published:** 2023-01-24

**Authors:** Jakob Kasper, Klaus Fiedler, Florian Kutzner, Chris Harris

**Affiliations:** 1 Department of Psychology, Heidelberg University, Hauptstraße 47, 69117 Heidelberg, Germany; 2 Department for Economic and Consumer Psychology, Seeburg Castle University, Seekirchen am Wallersee, Austria; 3 Department of Social, Health, Organizational Psychology, Utrecht University, Utrecht, The Netherlands

**Keywords:** Exploration/exploitation strategies, Positive testing, Metacognitive control, Sampling, Pseudocontingency

## Abstract

Why can initial biases persist in repeated choice tasks? Previous research has shown that frequent rewards can lure the decision maker into premature exploitation of a supposedly best option, which can result in the persistence of initial biases. Here, we demonstrate that even in the absence of rewards, initial biases can be perpetuated through a positive testing strategy. After eliciting a biased preference for one of two equally rewarding options, participants (*N* = 203) could sample freely from both options without the lure of any financial rewards. When participants were told to rule out alternatives in this phase, they explored the supposedly worse option and thereby managed to overcome their initial bias. When told to optimize their strategy, however, they exhibited a positive testing strategy resulting in the continued exploitation of the supposedly better option, a bias they maintained in an incentivized choice phase and later judgments. Across all participants, individual tendencies to exploit one option in earlier phases predicted biased behavior in subsequent phases. The findings highlight that not only the pursuit of instrumental rewards can lead to exploitation and the maintenance of initial biases. We discuss potential consequences for interventions.

## Introduction

The investigation reported in the present article elaborates on a recent conceptual integration of two fascinating issues: cognitive biases and illusions on the one hand and regulation processes along the exploitation versus exploration dimension on the other hand (Harris et al., 2020; Harris et al., 2023; Harris & Custers, In press). The basic idea is simple and straightforward, but nevertheless novel and replete with theoretical and practical implications. Cognitive biases or illusions are ubiquitous, but they are negligible as long as they remain transitory and can be corrected. Critically, however, they can translate into enduring fallacies, self-deceptions, and chronic mistakes with potential downstream consequences. Whereas prior research in the Kahneman-Tversky tradition was largely confined to fleeting biases such as first impressions in a momentary task setting (Tversky & Kahneman, 1974), a comprehensive approach to adaptive cognition must not only explain the occurrence of transitory biases in an initial stage but also their persistence during subsequent stages of critical assessment. What then makes it that some biases are immediately attenuated while others persist over time?

Recent research has mainly focused on how the hedonic value and instrumental rewards provided by outcomes influence whether biases will be transitory or whether they will endure (Denrell, 2005; Harris et al., 2020). For instance, in situations where frequent rewards inhibit exploration of the choice environment because individuals follow the maxim "never change a winning option," initial biases that would otherwise be detected may persist. In the current research, we argue that such an interpretation alone is too restrictive. Using a two-armed bandit task, we demonstrate that other processes such as positive testing (i.e., the general tendency to seek out hypothesis-confirming evidence rather than disconfirming evidence; Fiedler et al., 1999; Klayman & Ha, 1987), can lead to persisting biases even in the absence of instrumental rewards that could otherwise prompt biased behavioral tendencies. In doing so, we expand the exploration-exploitation account and highlight the importance of metacognitive monitoring and control processes for rational judgments and decisions (Ackerman & Thompson, 2017; Fiedler et al., 2019, 2021).

### Exploitation and exploration strategies

Judgments and decisions, in particular when they involve multiple alternatives or are dynamic in nature, require exploration of the various options before the benefits of superior alternatives can be exploited. Exploration also allows for metacognitive quality checks in the sense that hypothetical beliefs about the virtues and vices of certain aspects (locations, persons, action options) can be tested and resulting feedback can be experienced. This exploratory experience, however, comes with potential (opportunity) costs, and the time and effort expended in such exploration can no longer be used for exploitation of the action goals and for the consumption of rewards. Conversely, a rigid focus on exploitation of seemingly superior options leaves individuals vulnerable to local, premature optima and initial biases, and may keep them from finding even more profitable, global optima. Thus, individuals are constantly faced with the fundamental trade-off between acquiring more knowledge about environmental options and using that knowledge to maximize the options’ reward value (Fazio et al., 2004; Mehlhorn et al., 2015). Successfully balancing both strategies (exploration and exploitation) is crucial for optimal behavior regulation.

The basic paradigm applied in this study that captures the tradeoff between exploitation and exploration was first established in a series of experiments conducted by Harris et al. (2020). Participants could choose between two options, A and B, in a multi-trial two-armed bandit task. While the winning rates and expected payoffs were identical for both options, the outcomes during the first few trials were manipulated to create a bias in favor of one option, A. The initial bias was either induced by a genuine contingency within the first four observations (three wins for A vs. one loss for B) or by a pseudocontingency illusion such that winning and option A were more prevalent than not winning and B, but the relative winning rate was the same for A (9 out of 12) and B (3 out of 4). The alignment of two skewed distributions (with A as the more prevalent option and winning as the more prevalent outcome) causes an illusory (pseudo-)contingency (Fiedler, 2010; Fiedler et al., 2009, 2013) suggesting that A is superior to B. This is consistent with research showing that people have difficulties with the comparison of relative frequencies (see, e.g., the denominator neglect; Reyna & Brainerd, 2008).

Despite plenty of opportunities to put this initial pseudocontingency to the test during a subsequent extended sampling phase, the initial illusory bias favoring A was maintained in a reward-rich environment with a 75% winning rate for both options, such that the payoff structure of the sampling task rendered exploitation profitable. Only in a reward-impoverished environment with only 25% overall winning outcomes, which rendered exploitation frustrating, were participants motivated to switch from the supposedly better option A to the equally profitable option B and to get rid of the initial bias, induced by an illusory contingency favoring A.

Across several investigations, Harris et al. (2020, 2023) obtained consistent support for the notion that the tendency to exploit in a reward-rich condition prevented participants from correcting an initial bias. In contrast, reward-poor environments facilitated the tendency to explore new action opportunities and thereby to correct for initial biases. Apparently, then, the hedonic tone of the task setting and provided instrumental rewards function as chief moderators of adaptive behavior regulation. The indolence and apparent saturation resulting from frequent rewards seemed to motivate exploitation and counteract exploration, whereas the dissatisfaction resulting from infrequent reward and frequent frustration seemed to foster flexible learning.

### More than instrumental rewards

The aim of the present research was to extend and refine this interpretation of the regulation of exploitation and exploration strategies that points toward instrumental rewards as determinant for the stabilization or correction of cognitive biases, respectively. Note that in the extant literature, the regulation of self-control (Fujita et al., 2006) and of emotional responses (Gross, 1998) is typically conceived as a volitional, resource-intensive, hedonic process that depends on supportive payoff structures. Strategies are thought to be conscious intentions that require will-power and discipline, and successful regulation is often understood as an effortful process of sacrificing immediate desires and tolerating delay of gratification (Mischel et al., 1972).

However, such an instrumentally motivated view of regulation may be too restrictive. Exploitation or exploration strategies may be less constrained by social exchange principles or the balance of effort expenditure and motivating payoffs than expected. Rather, strategic shifts from exploration to exploitation, or vice versa, may be induced in manifold ways, some of which may appear insufficiently justified, purely incidental, or just reflective of human beings’ susceptibility to procedural priming, demand characteristics, or unconditional compliance with rules of social games.

It may be that in the context of exploration and exploitation strategies in particular, an effective experimental (or therapeutic) intervention does not depend on the payoffs in a reward-rich or in a reward-poor sampling phase. Perhaps the threshold for exploitation or exploration is much lower, devoid of any direct reward value. In the absence of monetary incentives, regulation may just as well be triggered by influences like confirmation of initially held beliefs (Pilditch & Custers, 2018) or the notorious human tendency to engage in positive testing (Klayman & Ha, 1987). Research on positive testing suggests a general tendency to search for hypothesis-confirming evidence more than for disconfirming evidence (Cameron & Trope, 2004; Fiedler et al., 1999; Klayman & Ha, 1987; Nickerson, 1998). Initial biased preferences for one of several options can influence subsequent information seeking, leading to different sample sizes of evidence (larger samples for initially preferred options), which alone is sufficient to affect individual judgments, even in the absence of instrumental rewards, cognitive distortions, or motivational influences (Fiedler et al., 1999).

In conclusion, incidentally induced exploitation and exploration strategies triggered by unmotivated and futile cues may moderate subsequent information sampling. Note that such a basic account serves to increase the causal weight given to easily solicited exploitation versus exploration strategies. It implies a much lower and less restrictive threshold for strategic moderation and a greater variety of experimental or therapeutic interventions required to influence exploitation versus exploration strategies. In case of biases caused by a one-sided exploitation focus, effective debiasing strategies that encourage an exploratory mindset may not rely on an outright redesign of the decision environment, for example, a manipulation of reward structures (see Harris et al., 2020; Harris et al., 2023; Harris & Custers, In press). Rather than altering instrumental incentives, we attempted to encourage exploration of a rarely encountered option by instructing participants to rule out alternatives that they perceived as inferior in a reward-free environment that rendered exploration costless.

### Outline of experimental paradigm

**Fig. 1:**
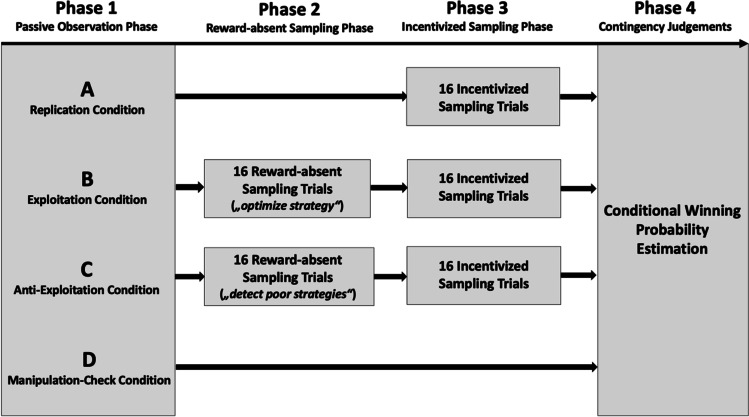
Schematic display of the experimental design employed for the present research. Participants were randomly assigned to one of four experimental conditions (A, B, C, D). Condition D only completed Phase 1 and Phase 4, while Condition A additionally completed Phase 3. Conditions B and C completed each experimental phase (1–4), albeit with different instructions in phase 2. Details are discussed in the text

For an empirical test of this extended conception, we developed a mixed design experiment consisting of four conditions (A, B, C, and D) exposed to different treatments across four procedural stages (see Fig. 1).

In the first phase, participants of all four conditions were exposed to a series of 16 passive observations intended to induce a pseudocontingency illusion in favor of one of two choice alternatives. We used the same double-skewed distribution of selected options and outcomes as Harris et al. (2020); that is, the same high (75%) winning rate held for the frequent option (9 wins out of 12) as for the infrequent option (3 wins out of 4). However, although this zero-contingency reflects two equivalent winning ratios for both alternatives, the alignment of two skewed base-rate distributions (skewed towards winning and towards the frequent option) creates the illusion of a higher winning rate of the frequent alternative (Fiedler, 2010; Fiedler et al., 2009; Fiedler & Freytag, 2004). The sole purpose of Condition D was a clean manipulation check. Immediately after the initial 16 observations, participants in this condition estimated the winning probabilities for both options, providing a check on the effectiveness of the initial induction of a cognitive illusion. In the other three conditions, no manipulation check was conducted to avoid self-consistency effects or committing participants to their explicitly stated initial preferences.

Condition A (replication condition) resembled the original task setting (in Experiments 2a and b) of the Harris et al. (2020) investigation for the reward-rich environment. Thus, without any further training or instructions, the participants in this condition continued to sample lottery outcomes (Phase 3 in Fig. 1), but now they could decide from which option they wanted to sample for each of the following 16 trials. If the outcome of the selected alternative was positive, they won 0.50€; if the outcome was negative, they lost 0.50€. Given this reward-rich environment (75% winning), we expected to replicate the Harris et al. (2020) finding that most participants would continue sampling from the frequent (and frequently winning) option and thereby fail to notice and correct for the illusory contingency.

The remaining conditions B and C underwent the same procedure but with one crucial difference. Prior to the incentivized sampling (with outcome-contingent rewards in Phase 3), participants in these two conditions could gather information about both options’ winning rates in a purely epistemic, reward-absent Phase 2. Instructions either encouraged (Condition B) or discouraged (Condition C) exploitation of the seemingly optimal option. Thus, independent of instrumental rewards, Phase 2 offered an incidental chance to exploit or to explore, respectively. The question of interest was to what degree this experience lacking instrumental rewards would influence subsequent sampling from the frequent option (exploitation) or a switch to the infrequent option (exploration) in an incentivized, reward-contingent sampling phase (Phase 3) as well as post-sequence conditional probability estimates of winning ratios (Phase 4).

## Methods

The experiment was run in the German language using the online survey platform SosciSurvey (Leiner, 2021). Participants were recruited via various social media platforms and advertisement at the Department of Psychology of Heidelberg University. The study was conducted in accordance with the guidelines of the ethics board of the faculty for behavioral and cultural studies at Heidelberg University. Before their participation, participants provided their informed consent.

### Transparency and openness

We report how we determined our sample size, all data exclusions, all manipulations, and all measures in the study. All data and analysis scripts have been made publicly available at https://osf.io/fjz3q/?view_only=40f6292f62a442d0bbffb1086c0887f4. The study design and analyses were not pre-registered.

### Participants and design

Overall, data were obtained from 203 participants (*N*_female_ = 118) with an average age of 26 years (*SD* = 11.87). To approximate an aspired statistical power of 1−*β* = 0.8 to capture even a rather small effect size at an 𝛼-level of 5% (*f*^*2*^ = 0.1; Harris et al., 2020), we estimated the required participant sample size to be n ~ 50 per condition. Participation was compensated either with course credit or with entry into a raffle with the chance of winning one of three 17€ vouchers. As mentioned above, informed consent was obtained at the beginning. The experiment included four conditions (see Fig. 1 for an overview).

### Materials and procedures

**Fig. 2:**
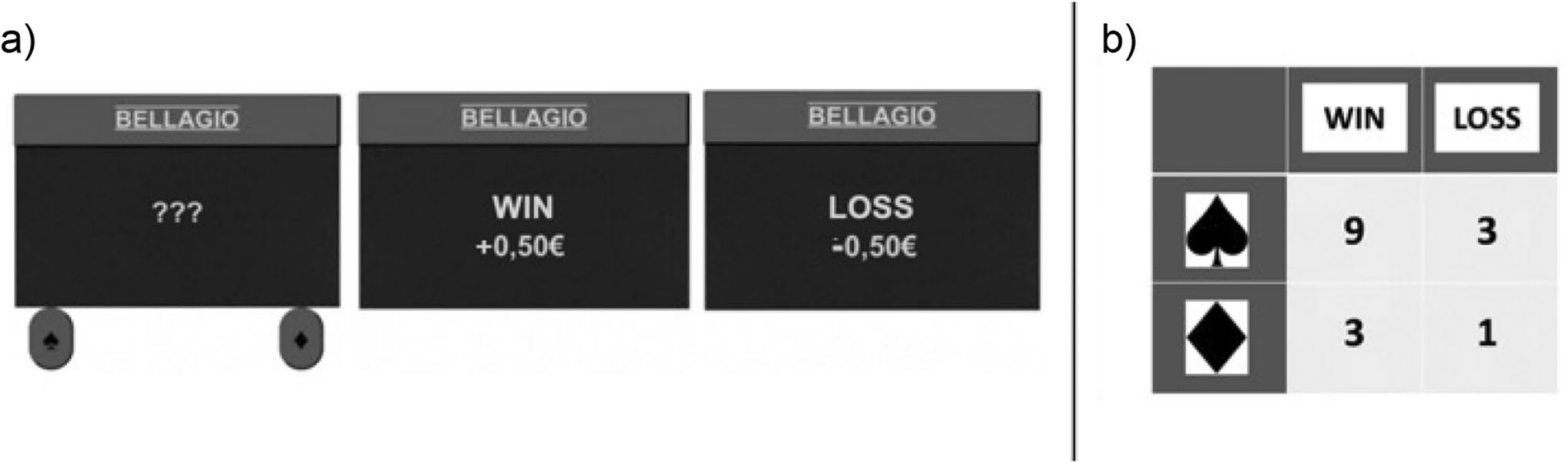
**a** Gambling machine “BELLAGIO” as it was presented to participants on any given trial before a button was pressed (???), and when displaying the feedback message for a win ("WIN + 0.50€") and for a loss ("LOSS - 0.50€"). **b** The double skewed joint distribution of buttons (spade/frequent button vs. diamond/infrequent button) and outcomes (win vs. loss) that was chosen to induce a pseudocontingency illusion of a higher winning rate for spades (frequent button) than for diamonds (infrequent button)

All instructions, stimulus displays, and dependent measures appeared on screen, in a computer dialogue. In all conditions, the initial instructions started with a description of the gambling machine “BELLAGIO” (see Fig. 2), resembling a typical two-armed bandit with two options (buttons “spades” and “diamonds”) and two outcomes (loss/win of 0.50€). Afterwards, all participants were exposed to a passive observation stage. We recorded a video of a random sequence of 16 trials played on the gambling machine (pointer clicking on either button followed by a display of the outcomes) that all participants viewed. Their task was to figure out the contingency between buttons and outcomes from the bivariate information of joint occurrence rates of buttons and outcomes. As all participants saw the same video, the same button was always presented frequently.

The 2 × 2 table on the right side of Fig. 2 gives the distribution of buttons and outcomes for the initial bias induction. The double-skewed distribution – 75% spades and 75% winning outcomes – was supposed to induce a pseudocontingency illusion favoring spades (frequent button) over diamonds (infrequent button), although the same 75% winning rate held for both options. Participants were informed that the winning rate for each button remained invariant throughout the entire experiment. In Condition D, the passive observation period was directly followed by the final conditional probability estimates. Participants estimated the winning rates for each button on a continuous scale from 0% to 100%, and also indicated their confidence on a scale from 0 to 100. A comparison of ratings for both buttons provided a manipulation check for the intended bias induction, which was left out for other groups to avoid self-consistency effects (Meiser et al., 2018).

In Condition A, the final incentivized sampling phase followed immediately after the initial bias induction phase (see Fig. 1). Participants were again informed that the winning odds for each option were the same as in the previous observation phase and could then choose between the two buttons in 16 incentivized sampling trials. They started with a capital of 13€ and were led to believe that the points they earned playing the gambling machine would determine the amount they could win in the raffle after the study if they chose to participate in it. The communicated payoff scheme was applied to motivate accuracy and win-maximizing strategies. However, since the actual sampling outcomes were not recorded by the programmed gambling machine, all participants who won the raffle following the study were rewarded with a 17€ voucher (expected outcome given that both gambling machine options led to a win of 0.50€ with a probability of 0.75 and to a loss of 0.50€ with a probability of 0.25). Thus, participants were only led to believe that the voucher amount was performance dependent. At the end of the 16-trial sequence, participants estimated the conditional winning probabilities for both options.

Between the initial observation and the subsequent incentivized sampling task in the last phase, participants in Conditions B and C completed a purely epistemic, reward-absent sampling phase. They again played 16 trials on the gambling machine. However, these trials would not create any payoff but merely served to inform an appropriate strategy for the following incentivized sampling phase. Accordingly, the gambling machine did not display specific values (€) but only showed whether the selected option would have resulted in a win or a loss. Consequently, the free exploration phase was independent of any payoffs or instrumental rewards. In Condition B (Exploitation), participants were instructed to consider the practice sampling trials of Phase 2 to gather further experience with the optimal choice strategy: “We therefore ask you to use these practice trials to test your approach for the subsequent phase and identify the best strategy possible!” In contrast, participants in Condition C (Anti-Exploitation) were told that research in game theory has shown that the best way to identify an optimal strategy was to rule out alternative ones. To induce such an anti-exploitation strategy, they were deliberately urged to try to perform as poorly as possible to determine which behavior to avoid in the final round: “We therefore ask you to try to perform as poorly as possible in the following practice trials. This will help you to develop a suitable approach for the actual game phase and to identify the best strategy possible!”

Recognizing that the initially induced bias favored the frequent button, participants in Condition C should feel encouraged to sample more from the infrequent option, facilitating a shift from exploitation to exploration, whereas participants in Condition B were expected to stick to exploiting the allegedly superior frequent option. Crucially, this mechanism should operate even in choice environments, where behavior was in no way influenced by instrumental rewards that could additionally foster exploitation otherwise. Because no payoff was provided on these practice trials in Phase 2, the chances of discovering the two buttons’ equal winning rates should be equivalent, although the practicing strategies should differ. Condition B should more often exploit the frequent button than Condition C. This reward-absent practicing stage in Phase 2 was again followed by an incentivized sampling period in Phase 3 and by the end-line conditional probability estimates in Phase 4 (see Fig. 1).

### Data preparation

Data preparation and analyzes were conducted with R (Version 4.0.4, R Core Team, 2019). Sampling behavior in the reward-absent and the incentivized sampling periods and conditional probability estimates provided the dependent variables. Therefore, sampled proportions of the infrequent and frequent button were used as simple measures of exploration and exploitation, respectively. The two measures that allowed us to assess perceived relative winning rates of the two buttons were the estimated winning probabilities for both buttons on a percentage scale from 0 to 100 (measured in Phase 4) and within-participant differences between those estimated winning rates for the two options, *Δp* = *p*(winning∣frequent button) – *p*(winning∣infrequent button). Positive *Δp*-scores indicate an advantage of the frequent button; negative *Δp*-scores suggest an advantage of the infrequent button; a zero score indicates no preference at all. Likewise, positive *Δc*-scores from confidence ratings indicate higher confidence for estimations of the frequent button, whereas negative scores reflect higher confidence for ratings of the infrequent button.

For the sampling behavior in Phases 2 and 3 and for the difference between winning probability estimates for each button (*Δp*) in Phase 4, we conducted two types of analyses. First, we used a set of *t*-tests to compare the sampling rates for the frequent button in Phases 2 and 3 and the difference between probability estimates (*Δp*) in Phase 4 against the absence of any biased preference for one of the two buttons, i.e., 50% in the case of sampling the frequent button in Phases 2 or 3 and *Δp* = 0 in the case of the difference between the probability estimates for both buttons, separately for Conditions A (Replication), B (Exploitation), and C (Anti-Exploitation). Second, we compared sampling rates for the frequent button in Phases 2/3 and the *Δp*-scores between conditions using targeted contrasts. Significance values were Holm-Bonferroni adjusted to account for multiple comparisons. In the Online Supplementary Materials (OSM), we report additional logistic regression analyses used to compare sampling of the frequent/infrequent button between conditions; results are consistent across both types of analyses.

For each analysis reported below, we excluded participants whose data for the specific analysis was below *q*_0.75_ + 1.5 * *IQR* or above *q*_*0.25*_ -1.5 * *IQR* (where *q*_0.75_ and *q*_*0.25*_ correspond to the first and third quartile of each experimental condition, respectively, and *IQR* is the difference between the third and the first quartile). Outliers were detected within experimental conditions and separately for each statistical analysis, such that participants were excluded only for specific analyses in which their values on one of the involved variables were considered statistical outliers, and no participant was completely excluded from the data set. Information on the number of participants that were excluded from each statistical analysis and a replication of each analysis without exclusions can be found in the OSM.

## Results

### Bias manipulation check

Recall that conditional probability estimates (winning rates for frequent vs. infrequent button; Phase 4) in Condition D afforded a manipulation check on the successful induction of an initial bias after Phase 1. Consistent with the expected pseudocontingency bias, participants in this condition provided higher winning estimates for the frequent (*M* = 67.46%, *SD* = 13.24) than for the infrequent button (*M* = 60.25%, *SD* = 19.14). The difference between probability estimates for both buttons, *Δp* = *p*(winning∣frequent) – *p*(winning∣infrequent), was significantly above zero (*M* = 7.21, *SD* = 21.49), *t*(47) = 2.32, *p* = .025, *d* = 0.34, indicating that the manipulation of an initial bias (used in all conditions) was apparently successful.

Confidence ratings for the frequent and infrequent button did not differ (*M* = 68.18%, *SD* = 21.71 versus *M* = 70.98%, *SD* = 25.58, respectively; *t*(48) = -1.11, *p* = 0.273, *d* = -0.16). Thus, there was no reason to include confidence in further analyses.

#### Sampling tendencies in the reward-absent sampling phase

**Fig. 3:**
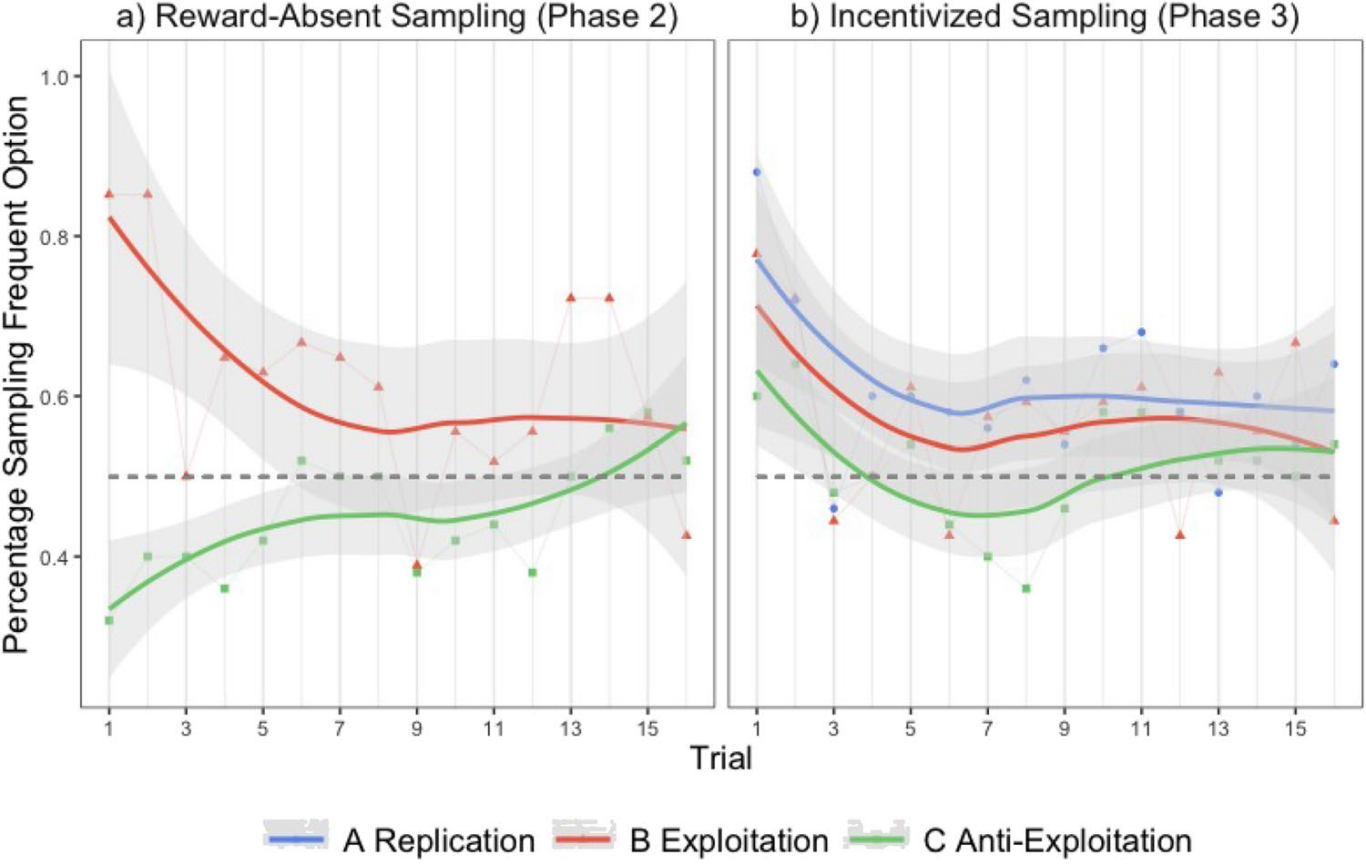
Mean sampling percentage of the frequent option by Conditions A (Replication), B (Exploitation), and C (Anti-Exploitation), separately for Phase 2 (reward-absent) and Phase 2 (incentivized), with trend lines separately fitted for Phase 2 and Phase 3

In the next step, we examined the sampling strategies in the reward-absent Phase 2, in which participants were guided to apply either an exploitation strategy (Condition B) or an anti-exploitation strategy (Condition C). We expected the rate of choosing the frequent button (i.e., rate of exploitation) to be higher in Condition B than in Condition C. Figure 3 (left chart) visualizes the pertinent results for Conditions B and C by illustrating the trial-by-trail average of participants exploiting the frequent option (separately for each condition) as an indicator of an exploitation strategy.

It is apparent that participants in Condition B, whose instruction encouraged exploitation, indeed exploited (*M* = 60.21%, *SD* = 11.94) more frequently than participants in Condition C (*M* = 45.41%, *SD* = 14.62), who were guided to use an opposite, anti-exploitation strategy, *t*(95) = 5.49, *p* < .001, *d* = 1.12. The above-chance (> 50%) preference for the frequent button in Condition B*, t*(51) = 6.17, *p* < .001, *d* = 0.86, was reversed in Condition C, *t*(44) = -2.10, *p* = .041, *d* = -0.31. Thus, the impact of an initially induced pseudocontingency bias on information sampling in a reward-absent, purely epistemic stage, depended on experimental instructions.

### Sampling tendencies in the incentivized sampling phase

Going one step further, the next question is whether the initially induced bias persisted during the incentivized sampling stage of Phase 3, in which correct choices were rewarded and incorrect choices were punished. As apparent from the right chart of Fig. 3, the vertical differences between the three curves were ordinally in line with the expected group differences, as elicited by the initial bias induction in Phase 1 and the opportunities to engage with the environment in Phase 2. While the tendency to exploit (choosing the frequent-button at a rate higher than 50%) during incentivized sampling in Phase 3 persisted in the Exploitation Condition B (*M* = 56.87%, *SD* = 15.13), *t*(49) = 3.23, *p* < .01, *d* = 0.46, there was no difference from 50% in the Anti-Exploitation Condition C (*M* = 53.55%, *SD* = 14.48), *t*(43) = 1.63, *p* = .111, *d* = 0.25. Yet, a direct comparison of exploitation rates between Conditions B and C rendered an insignificant result, *t*(136) = 1.08, *p* = .283, *d* = 0.22.

The pure Replication Condition A, in which no interim period of reward-absent sampling could have served to wash out the Phase 1 bias, exhibited the original exploitation bias to the strongest degree (*M* = 62.78%, S*D* = 15.13), *t*(44) = 5.66, *p* < .001, *d* = 0.84. This exploitation rate was significantly higher than in the Anti-Exploitation Condition C, *t*(136) = 2.92, *p* = .012, *d* = 0.62, but did not significantly differ from the Exploitation Condition B, *t*(136) = 1.93, *p* = .112, *d* = 0.40. The additional opportunity for exploration of the choice environment in the reward-absent sampling phase (Phase 2) seems to have somewhat reduced the tendency for exploitation in the following incentivized sampling phase (Phase 3) in both conditions (B, C), presumably reflecting a sort of saturation effect. The exploitation rate was reduced most in the Anti-Exploitation Condition (C).

### Conditional probability estimates

A very similar pattern characterized the final conditional probability estimates for both buttons, the difference of which, *Δp* = *p*(winning∣frequent) – *p*(winning∣infrequent), provided a measure of the perceived contingencies. Differences between end-of-sequence estimates *Δp* were significantly positive (showing persistent bias favoring the frequent button) in the Replication Condition A (*M* = 12.39, *SD* = 22.82), *t*(45) = 3.68, *p* < .001, *d* = 0.54, and the Exploitation Condition B (*M* = 6.02, *SD* = 15.99), *t*(50) = 2.69, *p* = .019, *d* = 0.38). In the Anti-Exploitation Condition C, the average *Δp* estimate was not significantly above zero, (*M* = 4.47, *SD* = 21.31), *t*(48) = 1.47, *p* = .149, *d* = 0.21. The focal contrast between Exploitation Condition B and Anti-Exploitation Condition C was not significant, *t*(143) = 0.39, *p* = .701, *d* = .08. The same goes for comparisons of the Replication Condition (A) with the Exploitation Condition (B), *t*(143) = 1.56, *p* = .244, *d* = .32, and the Anti-Exploitation Condition (C), *t*(143) = 1.92, *p* = .172, *d* = .39.

### Interim summary

Table 1 summarizes the evidence presented so far on the persistence of an initially induced bias across all stages of the longitudinal process. All table entries, except those for Condition C, retained the same sign as the initially induced bias favoring the more frequent option, despite repeated opportunities to correct this bias in the face of the reward-absent interaction with the choice environment in Phase 2 and instrumental learning in Phase 3. While Condition D afforded a manipulation check of the initial bias induction in a passive observation phase, detached from all subsequent active sampling, Condition A offered a perfect replication of Harris et al.’s (2020) findings for reward-rich environments. Condition B went even further, demonstrating that the initially induced bias was even more long-lived. It not only carried over to a single sampling stage as in Condition A, in which a high reward rate hedonically facilitated exploitation of the frequent option, following the wisdom “never change a winning option.” It even persisted when another reward-absent exercising phase, inserted prior to the incentivized sampling phase, rendered exploration costless and thus very easy. Although the bias shrunk somewhat from the reward-absent sampling in Phase 2 to the incentivized sampling in Phase 3, it continued to be significant in the preference to sample the frequent option in Phase 3 and in the contingency estimates in Phase 4.

**Table 1:** Summary of the impact of an initially induced bias favoring the Frequent Option on various dependent measures, as a Function of Experimental Conditions

Condition/Test	% sampling of frequent option in Phase 2	% sampling of frequent option in Phase 3	Δp difference between probability estimates for both options
Replication A	-	M = 62.78, SD = 15.13 t(44) = 5.66, d = 0.84	M = 12.39, SD = 22.82 t(45) = 3.68, d = 0.54
Exploitation B	M = 60.21, SD = 11.94 t(51) = 6.17, d = 0.86	M = 56.87, SD = 15.13 t(49) = 3.23, d = 0.46	M = 6.02, SD = 15.99 t(50) = 2.69, d = 0.38
Anti-Exploitation C	M = 45.41, SD = 14.62 t(44) = -2.10, d = -0.31	M = 53.55, SD = 14.48 t(43) = 1.63, d = 0.25	M = 4.47, SD = 21.31 t(48) = 1.47, d = 0.21
Manipulation Check D	-	-	M = 7.21, SD = 21.49 t(47) = 2.32, d = 0.34
Contrast B-C	t(95) = 5.49, d = 1.12	t(136) = 1.08, d = 0.22	t(143) = 0.39, d = .08

*Note:* The *t*-statistics and effect sizes *d* refer to contrasts of the mean dependent variable against 50% (in case of sampling the frequent button in Phases 2 or 3) and against 0 (in case of *Δp* measures of contingency estimates), calculated separately for Conditions A (Replication), B (Exploitation), C (Anti-Exploitation), and D (Manipulation check). The row Contrast B-C refers to the direct comparison between Condition B and Condition C

The overwhelming evidence for the persistence of an initial bias is contrasted by Condition C, in which an anti-exploitation strategy was induced in the reward-absent sampling Phase 2. Our instruction to sample the worst alternative led participants to focus on the supposedly worse option in Phase 2 (as evident from the negative sign of the t and d statistics in Table 1), and participants ultimately overcame their initial biases as is evident by the results of Phases 3 and 4. Note that the absence of any preference for one of the two options in Condition C is theoretically unsurprising because the higher base-rate of the supposedly superior button in the Phase 1 manipulation is cancelled out by the relatively higher base-rate of the supposedly inferior button in Phase 2. Consequently, the conditions for a pseudocontingency inference are no longer met. Individual participants may vary a lot in terms of their tendency to explore in Phase 2. Taking this interpersonal variation into account, it should therefore be possible to predict individual participants’ sampling of the frequent button (exploitation) in Phase 3, as well as their bias toward the frequent button in the final contingency estimates (Phase 4) from their free sampling behavior in Phase 2. Such an analysis is the purpose of the regression analyses reported in the next section.

### Process analysis

Logically, our approach rests on the causal assumption that the impact of an initial bias on any subsequent phase is contingent on the degree to which the same bias is conserved during intermediate phases. Only participants who maintained their pre-existing exploitation proneness in Phase 2 could be expected to stick to exploitation and fail to discover the equivalence of the infrequent option in the incentivized sampling in Phase 3 and Phase 4. Thus, individual exploitation rates in previous phases are the crucial predictor of the behavior measured in subsequent phases, not the aggregate exploitation rate per group.

For an adequate test taking inter-individual variation into account, we included both factors, assignments to experimental conditions B versus C and individual biases to favor the frequent option in preceding phases, as predictors in linear regression analyses of incentivized sampling rates in Phase 3 and contingency estimates in Phase 4. Including both group-level and individual-level biases as predictors in the same linear regression, the latter predictor should absorb the interpersonal error variance in the former, group-level predictor.

**Fig. 4:**
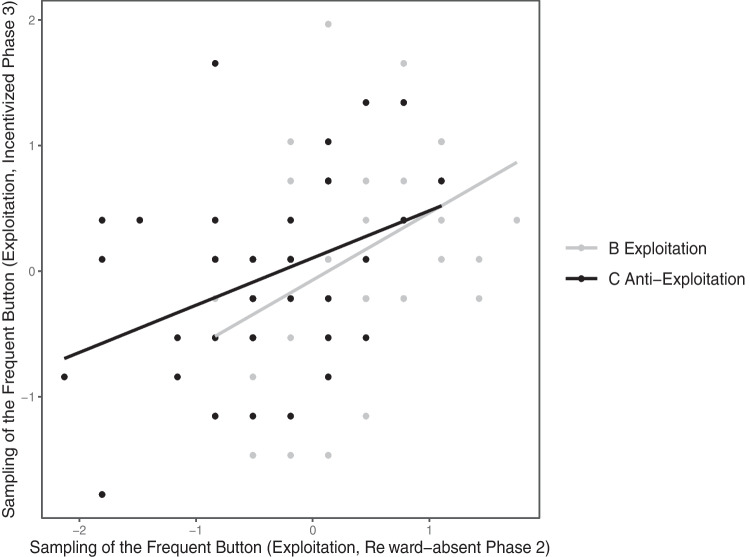
Mean *z*-standardized incentivized sampling of the frequent button (exploitation) in Phase 3 plotted as a function of the same individuals’ *z*-standardized sampling of the frequent button during the preceding reward-absent Phase 2. Grey and black dots (and regression lines) represent Conditions B and C, respectively

The regression results corroborate these expectations. Consider first the regression model for predicting the online measure of exploitation in Phase 3 from group assignment (B vs. C) and reward-absent sampling biases favoring the frequent button in Phase 2. Figure 4 displays the underlying two-dimensional plot, in which data points represent individual participants of Condition B (grey) and Condition C (black).

Whereas in Phase 2 (see Fig. 3) reward-absent sampling clearly differed between Conditions B and C (*r* = .52, *p* < .001), incentivized sampling in Phase 3 did not (*r* = .12, *p* = 0.244). The diminished group difference is evident from the close resemblance of both regression lines in Fig. 4. Yet, the slope of both regression lines is clearly positive, reflecting the dependency of individual biases in Phase 3 (vertical axis) on the sampling behavior in Phase 2 (horizontal axis). The corresponding zero-order correlation between reward-absent and incentivized sampling amounts to *r* = .41, *p* < .001.

We obtained distinct support for our rationale in a linear regression analysis, pooling over Conditions B and C. While the preceding sampling bias in Phase 2 significantly contributed to predicting the persistent bias in Phase 3, *β* = 0.56, *p* < .01, the impact of group assignment, *β* = 0.24, *p* = .301, and of the interaction, *β* = -0.17, *p* = .468, was negligible.

**Fig. 5:**
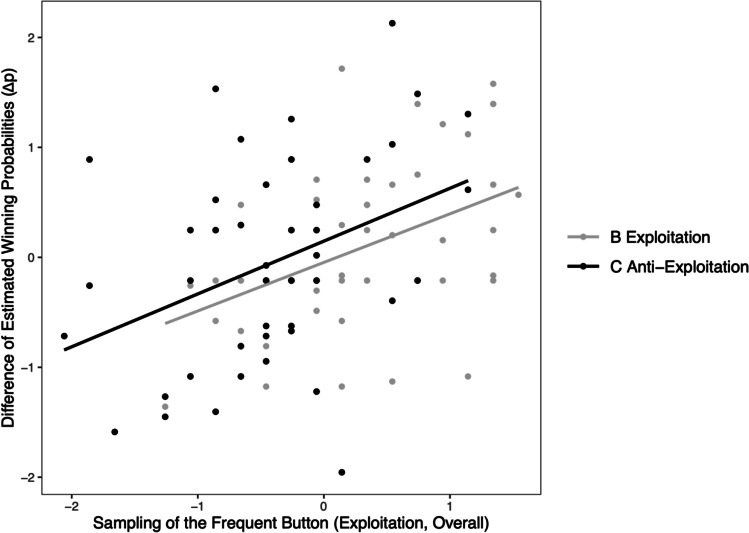
Individual (*z*-standardized) *Δp* scores (estimated winning probability for the frequent minus infrequent option in Phase 4) plotted as a function of (*z*-standardized) exploitation tendencies across Phases 2 and 3. Grey and black dots (and regression lines) represent participants of Conditions B and C, respectively

Plotting biases in the final contingency estimates as a function of biases in both preceding sampling Phases 2 and 3 yielded a similarly regular pattern of results (Fig. 5). Both regression lines are almost congruent, reflecting few differences between Condition B and C, but they exhibit the same positive slope. Individual-level biases in preceding phases were predictive of individual-level biases in final contingency estimates.

A regression analysis of Phase 4 contingency estimates corroborates this prima-facie interpretation. The tendency to exploit the frequent option across both preceding phases (2 and 3 combined) substantially predicted individual differences in the differences between final contingency estimates for both buttons (*Δp*) in Phase 4. The zero-order correlation between the individual-level predictor and the *Δp* estimates amounts to *r* = .39, *p* < .001; the corresponding regression weight is *β* = 0.42, *p* < .01. Group assignment (B vs. C), in contrast, hardly contributed to predicting the final estimates, *r* = .08, *p* = .446; *β* = 0.23, *p* = .291. The interaction of both predictors (*β* = 0.04, *p* = .864) was negligible. Both predictors, group assignment and individual sampling biases, correlated markedly, *r* = .44, *p* < .001.

Table 2 summarizes the linear regression results. It illustrates that, overall, the group-level factor (assignment to Condition B or C) was less predictive of individual biases in dependent measures than individual-level exploration versus exploitation differences in preceding phases. More regression details can be found in the Appendix.

**Table 2:** Summary of regression analyses of persistent biases favoring the frequent option as a function of experimental conditions and antecedent measures of individual biases

Criteria Predictors	Individual % reward-absent sampling of frequent option in Phase 2	Individual % incentivized sampling of frequent option in Phase 3	Individual Δp estimates reflecting bias favoring frequent option in Phase 4
Assignment to Condition B (+1) vs. C (–1)	r = .52 β = .52	r = .12 β = .24	r = .08 β = .23
Individual % reward-absent sampling of frequent option in Phase 2	-	r = .41 β = .56	r = .39 β = .42 (Pooling across Phases 2 and 3)
Individual % incentivized sampling of frequent option in Phase 3	-	-	

*Note.* Correlation and regression coefficients refer to previously reported regressions

## General discussion

Let us first summarize the findings obtained in the reported experiment with reference to the underlying theoretical considerations before we discuss broader implications for how to regulate biases in judgment and decision making.

### Summary of empirical evidence

The present experiment replicates and extends the Harris et al. (2020) findings in several notable respects. A deliberate manipulation check corroborates the premise that frequent exposure to one option in a reward-rich environment can lead to a pseudocontingency illusion favoring this option. The emergence of the illusory contingency due to the alignment of frequent winning and a higher prevalence of one option was confirmed by the estimated winning rates in the manipulation check. Participants judged the winning rate of the frequent option to be roughly 7.5% higher, although both options were equally rewarding (i.e., equal proportion of winning).

Further supporting the robust pattern discovered by Harris et al. (2020), as a reward-rich environment (i.e., a generally high winning rate) always fostered exploitation of the supposedly better option in subsequent sampling phases, the initial biases persisted tenaciously despite several opportunities to overcome the bias and recognize that the same high winning rate held for both options. Theoretically, the persistence of the initial bias is not surprising. As participants continued to exploit the frequent button, they generated again and again the conditions for a pseudocontingency illusion, namely, the alignment of a frequent option with frequent winning. As vividly illustrated in Fig. 3, a stable preference (> 50% sampling) was maintained as long as participants were not instructed otherwise.

The only effective countermeasure to overcome the initial bias was an exploration strategy. However, unlike previous experiments, we did not resort to a manipulation of the payoff rates to create reward-poor environments. Rather than facilitating a switch to exploration strategies through incentives – in the spirit of a win-stay-lose-shift rule (Nowak & Sigmund, 1993) – we resorted to a more subtle induction strategy. Holding the reward-rich environment constant, it was sufficient to rely on (a) encouraging choice strategies that led participants to consider the infrequent option in Condition C and experience the equivalence of both options in a playful, payoff-independent manner, and (b) spontaneous interpersonal variation in the tendency to exploit the frequent option. Note that Condition C constitutes a much weaker, less intrusive intervention that can be applied more realistically to real-world settings than the replacement of a reward-rich by a reward-poor reinforcement structure.

While Condition C succeeded in inducing a shift toward more exploration (preferential selection of the infrequent option), detached from any payoff, this strategy shift was not very strong and endurable. Unsurprisingly, when we reinstated the initial reward structure in Phase 3, participants on average no longer showed a preference for the infrequent option but regressed to a mixed strategy to sample both options to a similar extent. This result is in line with our theoretical framework, since the initial high base-rate of the frequent option in Phase 1 was countered by the exploration of the infrequent option in Phase 2, deleting the enabling preconditions of a pseudocontingency. It is unlikely that the reward-absent sampling of the infrequent option in Phase 2 could have led to a complete reversal of overall exposure rates to the two options. As a result, the reversal of preferences due to the anti-exploitation treatment of Condition C was confined to Phase 2 but did not induce more exploration in Phases 3 and 4.

Importantly, the failure of condition-wise means (Contrast B – C) to fully determine the expected pattern does not invalidate the rationale of our investigation, which predicts a persisting bias to the extent that individual participants actually exhibit an exploitation strategy in preceding phases. And, indeed, our regression analyses confirmed that individual-level tendencies to exploit in previous phases were the best predictor of a bias persisting in subsequent phases. A group-level predictor (assignment to Condition B vs. C) was regularly inferior to the individual-level predictor in these regression analyses (see Table 2).

To a certain extent our findings might also be explained in terms of a Bayesian framework. In particular, the bias induction and subsequent preference for the initially frequent option in Condition A would be expected in our pseudocontingency framework as well as from a Bayesian perspective. However, a Bayesian interpretation becomes more difficult for Conditions B and C: The instruction in Condition B to “… test your approach … and identify the best strategy possible” might still be interpreted from a Bayesian perspective to confirm the supposedly better (more frequent) alternative. However, the instruction in Condition C to “try to perform as poorly as possible…” in our eyes does not offer a clear Bayesian prediction. It is unclear whether this would result in sampling the frequent alternative (and hence reducing uncertainty for this option), sampling the infrequent alternative (and hence reducing uncertainty regarding possible alternatives), some combination thereof, or even other optimal behavior strategies. This is by no means meant to discredit a Bayesian framework, quite the opposite: We see the partial alignment (whenever clear predictions exist) of the Bayesian framework with the theoretical framework we laid out in this paper as important corroborative support. Nonetheless, in this particular setting our framework offers explanatory power above and beyond what a simple Bayesian updating rule could offer.

### Implications and practical advice

Theoretically and paradigmatically, these findings highlight the validity and stability of the role of exploration versus exploitation for the metacognitive detection and correction of everyday biases. It is well established that when feedback is choice-contingent, as is often the case, exploration of choice alternatives is necessary to update one’s beliefs (Cohen et al., 2004, 2007; Mehlhorn et al., 2015). Therefore, it is not surprising that providing opportunities to engage with rarely approached options has been seized as a frequently applied intervention strategy. For example, a canonical intervention to overcoming intergroup prejudices focuses on creating contact between groups – and thus opportunities for learning new information and updating one’s beliefs. The contact hypothesis (Pettigrew & Tropp, 2006) has often informed policy recommendations (Paluck et al., 2019; Paolini et al., 2021; Pettigrew & Tropp, 2006).

Critical to such applications and related translational research is the question of how to stimulate such adaptive exploration in practice. What interventions, instructions, payoff conditions, or learning opportunities are effective to render patients, students, consumers, or citizens open-minded and independent enough for exploration? The current research underlines that independent of reward-properties of a choice environment, an open-minded, exploratory mindset seems to be essential for metacognitive quality control, whereas exploitative approach strategies work against such adaptive regulation. In our study, promoting these mindsets did not require an outright redesign of the environment such as previously applied reductions in the overall reward rates of frequently approached options. As a matter of principle, exploration experiences were encouraged indirectly in an incidental, playful way, drawing on people’s general readiness to cooperate with the rules of any game or task.

However, the findings about Condition B suggests that offering low-risk exploration opportunities may not be sufficient to make interventions effective, whether or not there are material consequences (Fetchenhauer & Dunning, 2010). The subtle outside encouragement seemed to be a necessary adjunct for the intervention to effectively promote exploration and thus create opportunities to update initial, biased beliefs. Further, we have seen that those unsupported, incidentally induced exploration strategies may not be stable and may still give way to more exploitative strategies on many occasions. We should beware of the fact that short-term differences in the decision environment do not fully determine the trade-off between exploitation and exploration in the long term. Beyond the short-lived nature of the relative increase in exploration in the present Condition C, there are examples in which reward properties of an environment do not influence current behavior, for instance, reward-rich environments in which exploitation is neglected (Fiedler et al., 2021), or reward-absent environments that do not encourage exploration (Denes-Raj & Epstein, 1994).

Therefore, perhaps the most important practical advice to be gained from the present approach is that interpersonal variation is the key to adaptive regulation. We have seen that individual participants vary a lot in the extent to which they shift from exploitation to exploration. Translating this principle to variation within individuals, this suggests that maybe the most effective intervention is one that facilitates frequent shifts of strategies, and promotes methods like disconfirmation games, consider-the-opposite tasks, or devil’s advocate, rather than targeting one distinct behavior change. One specific example of a successful application of this principle is the Emphasis Change Training Method (Gopher et al., 1989; Yechiam et al., 2001), which was designed to improve learning in complex performance tasks by fostering exploration of alternative strategies during practice sessions. Such non-directive approaches to encourage exploration may be more effective than interventions attempting to (temporarily) force or oblige people to engage in a distinct regulation strategy, which may evoke reactance and the need to restore one’s freedom of choice and to engage in an opposite strategy. Exploration, in and of itself, may require freedom of choice and the allowance of self-determined variation between and within individuals.

## Appendix

### Detailed results of the illustrated regression analyses

Appendix Tables 3 and 4

**Table 3:** Regression Table – Regression Analysis 1

Predictor	Zero-order correlations	Regression weight (β)	SE	t-value	p-value
Intercept		-0.15	0.15	-0.98	.331
Group (C)	.12	0.24	0.23	1.04	.301
Exploitation (Phase 2)	.41	0.56	0.17	3.32	.001
Group x Exploitation (Phase 2)		-0.17	0.23	-0.73	.468

*Note*. Regression analysis of incentivized sampling of frequent button (exploitation, Phase 3) as criterion as function of group-assignment (B vs. C) and individual tendency towards reward-absent sampling of frequent button (exploitation) in the preceding Phase 2. Beta weights (*β*), confidence intervals (LL = lower limit; UL = upper limit), standard errors and significance tests for each predictor are displayed

**Table 4:** Regression Table – Regression Analysis 2

Predictor	Zero-order correlations	Regression weight (β)	SE	t-value	p-value
Intercept		-0.10	0.15	-0.69	.494
Group (C)	.08	0.23	0.21	1.06	.291
Exploitation (Phase 2 and 3)	.39	0.42	0.15	2.80	.006
Group x Exploitation (Phase 2 and 3)		0.04	0.22	0.17	.864

*Note.* Regression analysis of the *Δp*-score (differences between winning-probability estimates in Phase 4) as criterion as function of using exploitation of group-assignment (B vs. C) and overall exploitation rates. Beta weights (β), confidence intervals (LL = lower limit; UL = upper limit), standard errors (SE) and significance tests for each predictor are displayed
